# Stratified medicine in schizophrenia: how accurate would a test of drug response need to be to achieve cost-effective improvements in quality of life?

**DOI:** 10.1007/s10198-019-01108-4

**Published:** 2019-08-28

**Authors:** Huajie Jin, Paul McCrone, James H. MacCabe

**Affiliations:** 1 King’s Health Economics (KHE), Institute of Psychiatry, Psychology & Neuroscience at King’s College London, Box 024, The David Goldberg Centre, London, SE5 8AF UK; 2 The Department of Psychosis Studies, Institute of Psychiatry, Psychology & Neuroscience at King’s College London, London, SE5 8AF UK

**Keywords:** Schizophrenia, Stratified medicine, Health economics, Cost-effectiveness, Markov model, D61

## Abstract

**Objective:**

Stratified medicine refers to the use of tests that predict treatment response to drive treatment decisions for individual patient. The pharmacoeconomic implications of this approach in schizophrenia are unknown. We aimed to assess the cost-effectiveness of a hypothetical stratified medicine algorithm (SMA) compared with treatment as usual (TAU), for patients with schizophrenia who failed a first-line antipsychotic.

**Methods:**

A decision analytic model with embedded Markov process was constructed, which simulated the health and cost outcomes for patients followed SMA or TAU over a lifetime horizon, from healthcare and social care perspective. In the base-case analysis, sensitivity and specificity of the stratifier were both set as 60%. Extensive sensitivity analyses were conducted to test the impact of uncertainty around the value of important parameters. The primary outcome was the incremental cost per quality-adjusted life year (QALY) gained.

**Results:**

When both sensitivity and specificity of the stratified test were set at 60%, SMA appeared to be the optimal strategy as it produces more QALYs and incurs lower costs than TAU. This is robust to all scenarios tested. At a willingness-to-pay threshold of £20,000 per QALY, the probability for SMA to be the optimal strategy is 82.4%.

**Conclusions:**

Our results suggest that use of any stratifier with a sensitivity and specificity over 60% is very likely to be cost-effective comparing to TAU, for psychotic patients who failed a first-line antipsychotic. This finding, however, should be interpreted with caution due to lack of evidence for clozapine as a second-line antipsychotic.

**Electronic supplementary material:**

The online version of this article (10.1007/s10198-019-01108-4) contains supplementary material, which is available to authorized users.

## Introduction

Stratified medicine refers to the use of a stratifier (e.g. a biomarker or other predictive marker) to guide treatment choices. The mainstay of treatment for schizophrenia is antipsychotic medication, however, about one-third of patients with schizophrenia are treatment resistant (TRS)—they do not respond adequately to conventional antipsychotics other than clozapine [1]. Clozapine is the only drug with established efficacy in reducing symptoms and the risk of relapse for adults with TRS [2]. In addition, clozapine is also superior for negative and cognitive symptoms, as well as reducing suicidality and all-cause mortality [3]. Owing to safety concerns regarding the risk of neutropenia and agranulocytosis, the use of clozapine in most countries is restricted to patients who fail two non-clozapine antipsychotics at adequate doses [2, 4–6]. Although theoretically patients could be trialled on two non-clozapine antipsychotics in the space of a few months, in practice there is frequently a delay of several years before clozapine is prescribed [7]. There is emerging evidence, that ineffective antipsychotic treatment early in disease leads to worse long-term outcomes [8], and specifically that delay in prescribing clozapine to patients who are treatment resistant confers a poor outcome [9]. Thus, a stratifier which could predict TRS accurately and early might allow better decision making and lead to improved patients’ outcomes.

Significant efforts have been made to identify different stratifiers for predicting antipsychotic treatment responses for patients with schizophrenia, including immunological, neuroimaging and pharmacogenetic tests. However, the effect size of most stratifiers is small [10]. Recently, a large genome-wide association study conducted by the Chinese Antipsychotics Pharmacogenomics Consortium (CAPOC) showed that a combination of five novel loci could predict antipsychotic treatment response, with a sensitivity of 65% and a specificity of 69% [11]. It is therefore important to estimate whether using a test with this level of accuracy is sufficient to achieve cost-effective improvements in quality of life or not. This study aims to answer this question by estimating and comparing the long-term health and cost outcomes for patients who are treated with and without guidance of a hypothetical stratifier under different scenarios. The hypothetical stratifier can be any tests, such as immunological, neuroimaging or pharmacogenetic tests.

## Methods

This study was reported according to the CHEERS recommendations for reporting health economic evaluations [12].

### Population

The base case for the analysis consisted of a hypothetical cohort of adult patients with schizophrenia who failed a first-line conventional (i.e. non-clozapine) antipsychotic in the UK. It was decided that a stratifier would be more useful for patients who failed a first-line conventional antipsychotic than patients who are treatment-naïve, because the latter patient group has a high response rate to first-line conventional antipsychotic (ranges from 67.4 to 75.4% as reported by published literature [13, 14]).

### Competing treatment strategies

**Fig. 1:**
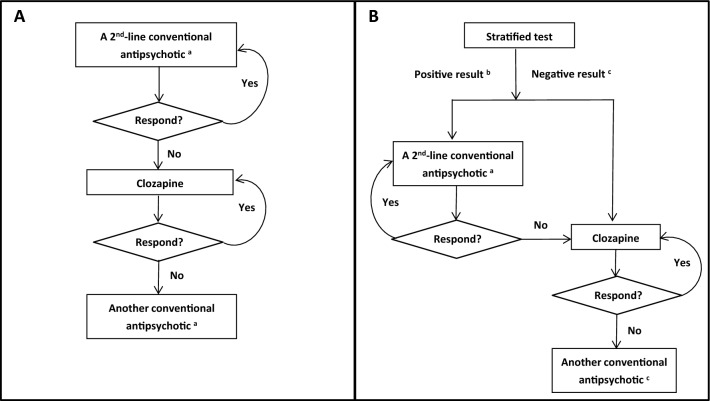
Protocol of treatment as usual (TAU) and stratified medical algorithm (SMA) for schizophrenia patients who failed a first-line conventional antipsychotic. **a** The conventional antipsychotics considered in the model include: olanzapine, amisulpride, aripiprazole, paliperidone, risperidone, haloperidol and flupenthixol decanoate. **b** Predicted to respond to a second-line conventional antipsychotic. **c** Predicted not to respond to a second-line conventional antipsychotic

Two treatment strategies were considered in the model: treatment as usual (TAU) and a stratified medical algorithm (SMA). Both strategies are described briefly below, and are visually shown in Fig. 1a, b, respectively. The primary outcomes of this analysis are lifetime cost and quality-adjusted life year (QALYs) gained for patients who received one of the alternative treatment strategies (TAU or SMA).

#### TAU

Patients would be treated according to the National Institute for Health and Care Excellence (NICE) schizophrenia guideline 2014 [2]. Thus, all patients who failed a first-line conventional antipsychotic would be treated with a second-line conventional antipsychotic. For this study, the seven antipsychotics included in the network meta-analysis conducted by the NICE schizophrenia guideline were modelled as second-line conventional antipsychotics, including olanzapine, amisulpride, aripiprazole, paliperidone, risperidone, haloperidol and flupenthixol decanoate [2]. Patients who did not respond to a second-line antipsychotic would be switched to clozapine; while patients who did not respond to, or experienced an intolerable adverse reaction to clozapine, would be switched to another conventional antipsychotic.

#### SMA

All patients who failed a first-line conventional antipsychotic would undergo a stratified test designed to predict their response to second-line conventional antipsychotics. Patients predicted to be AP2 responders (positive test result) would be switched to a second-line conventional antipsychotic; while patients predicted not to be AP2 responders (negative test result) would be switched immediately to clozapine, thus bypassing the second treatment trial. Patients who failed a second-line conventional antipsychotic would be switched to clozapine; while patients who failed clozapine would be switched to another conventional antipsychotic.

### Key impacts of the stratifier modelled in the study

In order to estimate the lifetime impact of the stratifier on total cost and QALYs, the following impacts of using a stratifier were modelled: clinical benefits, clinical harms, cost of stratifier, and cost impact on other resource use. A brief description of each impact is presented below. The clinical benefit of using SMA considered in the model is that clozapine responders who are correctly identified as AP2 non-responders (true negative) can initiate clozapine once they fail a first-line antipsychotic, without having to try an ineffective second-line antipsychotic first. Therefore, the costs saved from treating otherwise relapsed patients, the increase in QALYs arising from spending less time in a state of relapse, and their reduced mortality accruing from the use of clozapine, were modelled. The potential harm of using SMA is, if the stratifier is not 100% accurate, some AP2 responders might be wrongly identified as AP2 non-responders (false negative) and switched to clozapine unnecessarily. AP2 responders who were wrongly predicted to be AP2 non-responders (false negatives), and were thus prescribed clozapine, would have a 71.16% probability of responding to clozapine and therefore remain on it [13, 15]. The remaining 28.84% patients who do not respond to clozapine would be offered a second-line conventional antipsychotic, which they would respond to. Compared to most conventional antipsychotics included in the model, clozapine is associated with higher adherence rate and lower incidence of acute extrapyramidal symptoms (EPS), but is more likely to cause other adverse reactions, including weight gain and diabetes [2]. Furthermore, patients on clozapine are at risk of neutropenia and thus need to be monitored frequently. There is therefore a cost to being unnecessarily prescribed clozapine, which was included in the model.

### Model structure

**Fig. 2:**
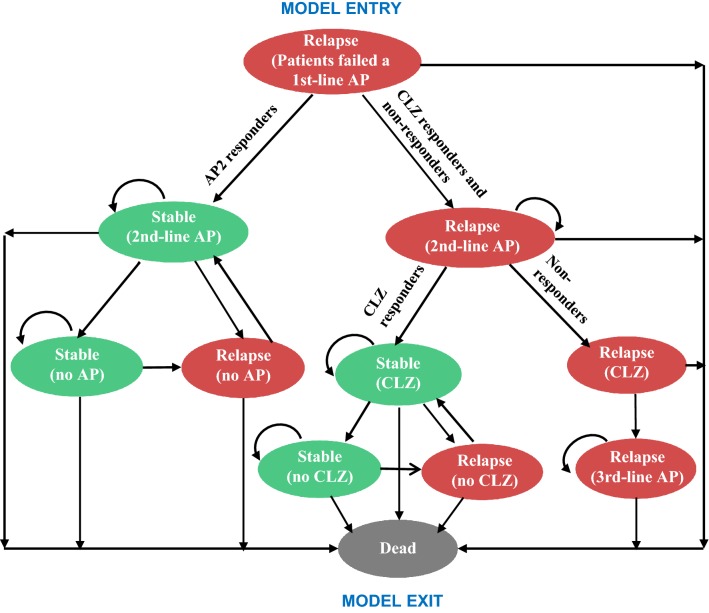
State transition diagram of the Markov model for patients in the TAU arm. *AP* conventional antipsychotic, *CLZ* clozapine

**Fig. 3:**
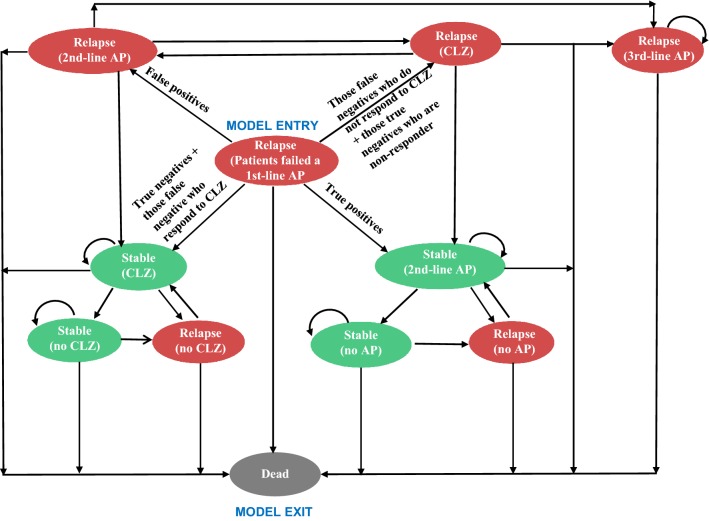
State transition diagram of the Markov model for patients in the SMA arm. *AP* conventional antipsychotic, *CLZ* clozapine

In order to capture the key impacts of using a stratifier (as described above), a decision analytic model with a Markov process embedded was developed using TreeAge 2014 (TreeAge Software, Williamstown, MA). Within the Markov process, events of interest are modelled as transitions from one health state to another. The time period of the model is divided into cycles of time, for example a year for this study, and at each cycle, probabilities are assigned of remaining in the same state or moving to a different state within the model. A 1-year cycle was chosen because most antipsychotic discontinuation happened within a year after the initiation of a new antipsychotic [16]. The economic model built for the NICE schizophrenia guideline also adopts a 1-year cycle [2]. In our model, eleven mutually exclusive health states were included. The transition between different health states for patients in the TAU arm and the SMA arm are illustrated in Figs. 2 and 3, respectively. Which health state the patient would be in depends on the patient’s subgroup (AP2 responder, clozapine responder, or non-responder) and which antipsychotic treatment the patient is currently on (conventional antipsychotic, clozapine, or no antipsychotic due to non-adherence). Each of the health states is assigned a cost and effectiveness value that patients accrue while in that state. For each treatment, the overall costs and effectiveness are calculated on the basis of the total length of time patients spend in each health state over the time horizon. Both costs and QALYs were discounted at an annual rate of 3.5%, as recommended by NICE [17], to reflect society’s preference for costs to be incurred in the future rather than the present, and for benefits to be experienced in the present rather than the future. Use of antipsychotics are associated with a wide range of adverse physical and mental health events. Based on the incidence rate, magnitude of health and cost impact and data availability, four adverse events of antipsychotic medications were selected for this model: weight gain, acute EPS, diabetes and neutropenia (for patients on clozapine only).

It was assumed that within the target population, there are three patient subgroups:

- AP2 responder, patients who will respond to a second-line conventional antipsychotic.
- Clozapine responder, patients who will fail to respond to a second line antipsychotic but will subsequently respond to clozapine.
- Non-responder, patients who will not respond to any existing antipsychotics including clozapine.

Wherever a patient responded to an antipsychotic, it was assumed that they would continue to receive the same antipsychotic in the following cycles. Patients who responded to a second-line antipsychotic but did not adhere to their medication were assumed to remain without antipsychotic treatment until they experienced a relapse, after which they were assumed to return to the same antipsychotic.

### Input data

Cost-effectiveness analysis requires four types of data: clinical evidence, health-related preferences (utilities), healthcare resource use and costs. A summary of the key parameters used in the model is reported in Table 1 and briefly described below; while a detailed description of all parameters used in the model is reported in Online Resource 1. In the base-case analysis, both the sensitivity and specificity of the stratifier were set at 60% (similar to the accuracy data reported by the study conducted by CAPOC [11]), with a range of 0–100% was tested in sensitivity analyses. There is a lack of clinical evidence about patients’ response to a second-line antipsychotic after failing a first-line antipsychotic. According to a recent literature review [14], there are only two trials which examined patients’ response to a second-line antipsychotic after failing a first-line antipsychotic: one is a naturalistic study conducted by Agid et al. [13], and another is an open-label treatment with clozapine in first-episode schizophrenia and schizophreniform disorder (OPTiMiSE) trial [14]. It was decided that the data reported by Agid et al. is more appropriate for this model for two reasons: (1) Agid et al. has a larger sample size than the OPTiMiSE trial (60 vs 39, number of patients who were switched to a second-line antipsychotic after failing a first-line antipsychotic); (2) The results of the OPTiMiSE trial indicated that for patients who did not achieve remission with a first-line antipsychotic within 4 weeks, switching to a second-line conventional antipsychotic did not improve their response rate—or in another word, for patients who failed a first-line antipsychotic, the proportion of AP2 responder is nearly zero. The lower the proportion of AP2 responder, the higher the proportion of clozapine responder and non-responder, and the more cost-effective the stratifier, as the key benefit of using the stratifier is to help clozapine responders to initiate clozapine earlier compared to the current practice. Therefore, to be conservative about the potential benefits of using the stratifier, the data reported by Agid et al. was used in the base case analysis, while a different range of data was tested in sensitivity analyses. Based on the data reported by Agid et al., it was calculated that for patients who failed a first-line antipsychotic, 16.67% of them responded to a conventional second-line antipsychotic (risperidone or olanzapine was used in the study), 62.50% responded to clozapine, and 20.83% did not respond to any antipsychotic tested in the study. Therefore, in the model, it was assumed that the proportion of different patient subgroups are: AP2 responder (16.67%), clozapine responder (62.50%) and non-responder (20.83%). The proportion of positive and negative test results depend on the sensitivity and specificity of the stratifier, and the proportion of AP2 responders. Under a stratifier with 60% sensitivity and specificity, and assuming 16.67% of target population are AP2 responders, 43.33% patients will get positive results (10.00% are true positive and 33.33% are false positive), while 56.67% patients will get negative results (50.00% are true negative and 6.67% are false negative). Patients’ probability of non-adherence to conventional antipsychotics and probability of developing adverse events were obtained from the network meta-analyses reported in the NICE schizophrenia guideline [2].

**Table 1:** Summary of key parameters used in model

Parameters	Base-line value	Range tested in one-way or two-way sensitivity analysis		Distribution			Source	
Diagnostic efficacy of predictive test								
Sensitivity (proportion of second-line antipsychotic responders that are correctly identified as such)	0.60	0–1.00		Assumed fixed			Estimate of what may be achievable	
Specificity (proportion of second-line antipsychotic non-responders that are correctly identified as such)	0.60	0–1.00		Assumed fixed			Estimate of what may be achievable	
Distribution of patients who failed a first-line antipsychotic by subsequent response/non-response								
Clozapine responder	62.50%	0%–79.15%		Dirichlet distribution (*n* = 21.0)			Agid et al. [15]	
AP2 responder	16.67%	N/A		Dirichlet distribution (*n* = 5.6)			As above	
Clozapine non-responder	20.83%	N/A		Dirichlet distribution (*n* = 7.0)			As above	
Response to different antipsychotics in misclassified individuals								
AP2 responder’s response to clozapine	71.16%	0–1		Beta distribution (SD assumed to be 50% of mean value)			Calculated from Agid et al. [15] and McEvoy et al. [1]	
AP2 non-responder response to second-line antipsychotics	0%	N/A		Assumed fixed			Estimate	
Cost data								
Cost of predictive test			£500.00		£100.00–1,000.00	Gamma distribution (SD assumed to be 50% of mean value)		Estimate

Interventions	Base-line value	Range tested in one-way or two-way sensitivity analysis	Distribution	Source
Annual cost of treating patients with active psychosis	£39,141	N/A	As above	Uplifted from the NICE schizophrenia guideline [2]
Annual cost of treating remitted patients	£15,086	£10,000–£39,141	As above	As above
Health-related quality of life data				
Relapse	0.4790	0.1900–0.6040	Beta distribution (SD: 0.0330)	Briggs et al. [24]
Stable schizophrenia	0.8650	0.8650–0.9190	Beta distribution (SD: 0.0210)	As above
Other input data				
Annual discount rate for both costs and outcomes	0.0350	0–0.050	Assumed fixed	NICE guideline manual [19]
Cycle length	1 year	N/A	Assumed fixed	Estimate
Number of cycles	80	10–100	Assumed fixed	Estimate

A complete list of all parameters used in the model is reported in Online Resource 2, Table 1

Utility data were obtained from a UK study which reported separate utility scores for stable or relapsed schizophrenia patients with or without adverse events of antipsychotics [18]. For costing data, the model took the perspective of the UK health and social care system, as recommended by NICE [17]. All costs were reported in 2017 UK pounds. The cost of the stratifier was assumed to be £500 per patient (an estimate of the cost of a magnetic resonance spectroscopy scan, which is considered to be the higher end of the likely cost of a stratifier) with a range of £100–1000 tested in sensitivity analyses [19]. Unit costs were based on the NHS reference costs 2016–17 [20], prescription cost analysis (England) 2017 [21] or the Unit Costs of Health and Social Care 2017 [22]. Resource quantities were mainly informed by the NICE schizophrenia guideline 2014 [2].

### Sensitivity analysis

The credibility of the results of economic analysis largely relies on the validity of input data. Sensitivity analyses can test the effect of changes in the input data on the observed results. If, after performing sensitivity analyses, the findings are consistent with those from the baseline analysis and would lead to similar conclusions about the cost-effectiveness of different strategies, we can be reassured that the uncertainty of input data has little impact on the primary conclusions. For this study, three types of sensitivity analyses were conducted: one-way sensitivity analysis which assess the impact of uncertainty around the value of each individual parameter, two-way sensitivity analysis which assess the impact of uncertainty around the value of two correlated parameters (e.g. sensitivity and specificity of the stratifier), and probabilistic sensitivity analysis (PSA) which examine the impact of joint uncertainty of multiple parameters simultaneously. A summary of all parameters tested in sensitivity analysis is reported in Online Resource 1, Table 1.

### Model verification and validation

A summary of the activities conducted for model verification and validation is presented in Table 2.

**Table 2:** Verification and validation process and people involved

Verification and validation process	People involved
1. Check appropriateness of the model structure	JM and PM
2. Check appropriateness of data source	JM and PM
3. Compare data used in the model against the evidence sources	HJ
4. Check model logic (white-box testing)	HJ
5. Check the plausibility of the intermediate and final outputs, including results of sensitivity analyses (black-box testing)	HJ, JM and PM
6. Compare results with published literature	HJ

*HJ* Huajie Jin, *JM* James MacCabe, *PM* Paul McCrone

## Results

**Fig. 4:**
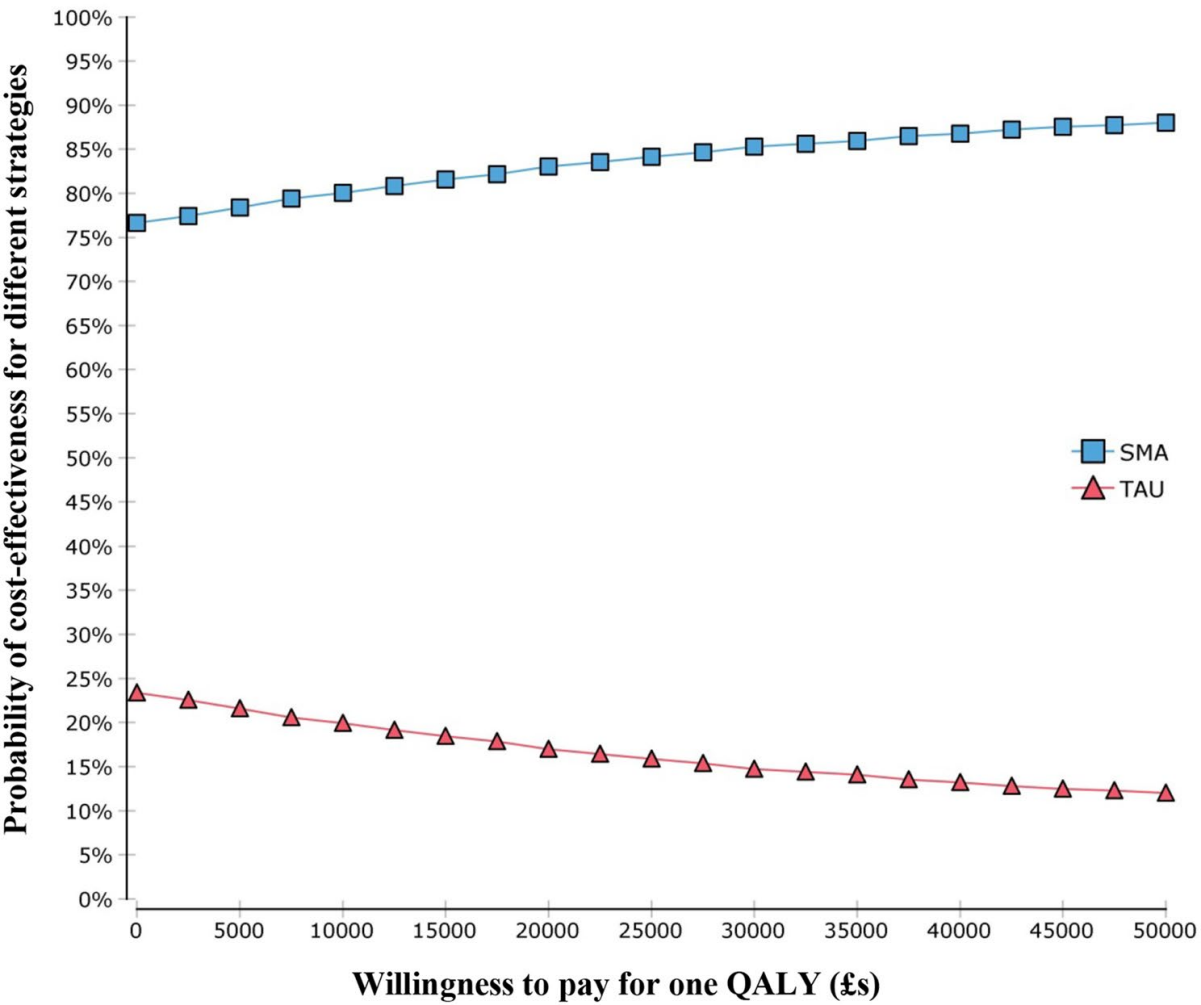
Cost-effectiveness acceptability curves showing probability that each strategy is most cost-effective option at different willingness-to-pay (WTP). *SMA* Stratified medicine algorithm. *TAU* Treatment as usual

The base-case analysis results are presented in Table 3. Compared to TAU, use of the SMA produced 0.10 more QALYs and incurred £7363 lower costs per person. The results of one-way and two-way sensitivity analysis are reported in detail in Online Resource 2 and is summarised below. One-way sensitivity analysis showed that our conclusion (SMA is more cost-effective than TAU) was robust to all parameters tested. Two-way sensitivity analysis shows that even when the sensitivity of the stratifier is 0%, as long as the specificity of the stratifier is no less than 11.50%, SMA is still more cost-effective than TAU. If the sensitivity of the stratifier is 50%, as long as the specificity of the test is no less than 6%, SMA is more cost-effective than TAU. The result of PSA is shown in Fig. 4. If the NHS was not willing to attach any monetary value to QALY gains, the likelihood for SMA to be the most cost-effective option is 76.62%. As a unit improvement is valued at higher levels this likelihood increases. At a WTP threshold of £20,000 per QALY, there is a 83.04% likelihood that SMA is the most cost-effective option.

**Table 3:** Incremental costs and QALYs per person by treatment strategy

Strategy	Total Costs (£)	Total QALYs	Incremental cost (£)	Incremental QALYs
TAU	510,239	16.15	–	–
SMA	502,876	16.25	− 7363	0.10

## Discussion

### Main findings and interpretation

Significant efforts have been made to identify stratifiers for predicting antipsychotic treatment responses for patients with schizophrenia. However, little is known about how accurate such a stratifier needs to be to achieve cost-effective improvements in quality of life. To the best of our knowledge, only one previous study has assessed the cost-effectiveness of a stratifier for schizophrenia patients, which is a modelling study conducted by Perlis et al. [23]. The stratifier assessed was a genetic test that can identify individuals with greater likelihood of responding to second-line conventional antipsychotics, with a sensitivity of 95.9% and a specificity of 38.3%. Perlis et al. found that applying the test to treatment-naïve patients and using clozapine as a first-line antipsychotic for those predicted to respond to clozapine is more effective but also more expensive, comparing with no test and reversing clozapine as a third-line antipsychotic. However, the study conducted by Perlis et al. had some limitations: (1) it did not consider the health or cost impact of any adverse events of antipsychotics, which might have important impacts on the cost-effectives results; (2) PSA was not conducted to examine the impact of joint uncertainty of multiple parameters simultaneously; (3) this study was published in 2005, therefore, it has limited relevance to the current context, due to rapidly changing nature of treatments, health services, evidence base and unit costs. No previous studies have assessed the cost-effectiveness of a stratifier for schizophrenia patients who fail a first-line antipsychotic. In order to fill the gap, we used decision-analytic modelling to estimate the cost-effectiveness of a SMA for schizophrenia patients who failed a first-line antipsychotic, over a life-time time horizon from the health sector and social care perspective. Results indicate that the stratified medicine algorithm was more cost effective that treatment as usual, when both the sensitivity and specificity of the stratifier was set at 60%. One surprising finding of this study is, even when the sensitivity of the stratifier is set to 0%, as long as the specificity of the stratifier is no less than 11.50%, SMA is still more cost-effective than TAU. This was mainly because the proportion of patients who would respond to clozapine is much larger than the proportion of patients who would only respond to a second-line conventional antipsychotic. A 0% sensitivity means none of the AP2 responders are correctly identified as such—all of them are identified as AP2 non-responders and therefore be prescribed with clozapine. According to Agid’s study [13] and the CATIE trial [15], 16.67% of the target population are AP2 responders; and 71.16% of AP2 responders would also respond to clozapine. This means, only those AP2 responders who do not respond to clozapine (16.67%*(1–71.16%) = 4.81%) are adversely affected by the poor sensitivity of the stratifier, and even then, they are only on clozapine for 1 year in our model, before being switched to a non-clozapine antipsychotic, to which they would respond. For those AP2 responder who also respond to clozapine (16.67%*71.16% = 11.86%), our model shows that although they are slightly better-off with a conventional second-line antipsychotic comparing to clozapine, the overall magnitude of cost and QALY difference is very small. The specificity of the test, in this model, is its ability in correctly identifying AP2 non-responders, which includes clozapine responders and non-responders. According to Agid’s study [13], clozapine responders comprise 62.50% of the target population. A 11.50% specificity means, comparing to TAU, 7.19% (= 11.50%*62.50%) of the target population received the right treatment (clozapine) one cycle earlier, and therefore results in lower cost and higher QALY gains. To sum up, comparing to TAU, use of a stratifier with 0% sensitivity and 11.50% specificity results in worse outcomes for 4.81% patients (AP2 responders wrongly identified as clozapine responders, and who do not respond to clozapine) and better outcomes for 7.19% patients (clozapine responders correctly identified as AP2 non-responders). Therefore, after weighing up the clinical benefits, clinical harms and cost implications of the stratifier, SMA is considered to be more cost-effective than TAU.

According to a recent systematic review [14], there are only two trials which examined patients’ response to a second-line antipsychotic after failing a first-line antipsychotic: Agid et al. [13] and the OPTiMiSE trial [14]. Both studies found that for patients who failed a first-line conventional antipsychotic, their response to a second-line conventional antipsychotic is poor—in fact, the OPTiMiSE trial found that for patients who did not achieve remission with a first-line antipsychotic within 4 weeks, it might be better for them to continue their current antipsychotic, rather than being switched to a second-line conventional antipsychotic. The findings of these two studies, combined with the result of our model (a stratifier with very low sensitivity and specificity is still considered to be cost-effective) raises the question of whether a stratifier is necessary for patients who failed a first-line antipsychotic or not—using clozapine as a second-line treatment without any stratifier might turn out to be more cost-effective, compared to use of a stratifier with low sensitivity and/or specificity. Although a few studies have argued the potential benefits of using clozapine as a second-line or even first-line treatment [24–26], there is currently a lack of high-quality evidence about clozapine’s efficacy for schizophrenia who failed a first-line antipsychotic. Further research is urgently needed to establish clozapine’s clinical efficacy and safety profile for patients as a second-line treatment. With publication of such results, the input data of the present analysis can be updated to shed light on whether clozapine should be used as a second-line antipsychotic.

### Strengths and limitations

To our knowledge, this is the first study to explore the pharmacoeconomic implications of using a stratified medicine approach in schizophrenia patients who failed a first-line antipsychotic. A major strength of this study was the collective use of the state-of-the-art methodology and most up-to-date data to evaluate the cost-effectiveness of a hypothetical stratifier. Decision analytic modelling provides a framework to quantify and synthesize the clinical benefits, clinical harms and cost implications of the stratifier. Comprehensive sensitivity analyses have been conducted to test the uncertainty of the result. Therefore, commissioners and clinical researchers can be reassured that the conclusion of this paper is robust to large changes or errors in the input parameters.

There are a number of limitations of the economic model presented here, the majority of which derive from limitations in the evidence base used to populate the model. For example: (1) patients’ responses to second-line antipsychotics were calculated based on the data for 60 schizophrenia patients included in the Agid et al. [13], as there is a lack of larger trials which specifically assessed patients’ responses to a second-line antipsychotic after failing a first-line antipsychotic. There is uncertainty about the generalizability of the results reported by Agid et al. due to its small sample size; (2) patients’ probability of developing adverse events were obtained from the network meta-analyses reported in the NICE schizophrenia guideline [2]. It was noted that the population included in those network meta-analyses were ‘general’ schizophrenia patients in remission, some of whom might have been on antipsychotics for many years. It is unknow whether those patients would have different probability of developing adverse events, compared with our target population. However, in this study, the limitation related to parameter uncertainty has been partially mitigated by extensive sensitivity analyses. Secondly, discontinuation of antipsychotics due to intolerable adverse events was only modelled for clozapine, but not for other conventional antipsychotics. This simplification is against the use of clozapine, and thus against the use of stratifier, as the key benefit of using the stratifier is that clozapine responders who are correctly identified as AP2 non-responders can initiate clozapine sooner than the current practice. The base case results show that even when this simplification was used, SMA still dominates TAU, which demonstrates the robustness of our base case conclusion. Thirdly, the cost and health impacts of adverse events of antipsychotics have not been fully captured. Other than the four adverse events modelled in this study (i.e. weight gain, diabetes, acute EPS and neutropenia), there are other adverse events which might impact on patients’ QALY and cost outcomes, such as tardive dyskinesia, sexual dysfunction, increase in prolactin levels, and agranulocytosis. Even for those adverse events which were considered in the model, the impacts of complications of those adverse events were not fully captured: for example, the impact of complications of diabetes (e.g. myocardial infarction and stroke etc.) on mortality were not considered in the model. Fourthly, besides the health and social care costs that were considered in this analysis, SMA is likely to have an impact on the wider societal costs (such as costs borne to the criminal justice system and productivity losses for schizophrenia patients and their carers). When wider societal costs were considered, the gap between treatment cost of remitted patients and relapse patients is likely to widen [27], and thus making SMA more cost-effective.

## Conclusion

Our results suggest that use of any stratifier (e.g. immunological, neuroimaging or pharmacogenetic tests) with a sensitivity and specificity over 60% is very likely to be cost-effective comparing to TAU, for psychotic patients who failed a first-line antipsychotic. This finding, however, should be interpreted with caution due to lack of evidence for clozapine as a second-line antipsychotic.

## Electronic supplementary material

Below is the link to the electronic supplementary material.

Supplementary material 1 (PDF 551 kb)

## Data Availability

All data generated or analysed during this study are included in this published article and its electronical supplementary martial (Online Resource 1–2).
