# Supplementary material for: Stratified medicine in schizophrenia: how accurate would a test of drug response need to be to achieve cost-effective improvements in quality of life?
Source: Eur J Health Econ. 2019 Aug 28;20(9):1425–35. doi: 10.1007/s10198-019-01108-4 (PMC6856032; doi:10.1007/s10198-019-01108-4)
Supplement: Supplementary file 1 — Supplementary material 1 (PDF 551 kb) [file 10198_2019_1108_MOESM1_ESM.pdf]

1. Title of paper

Stratified medicine in schizophrenia — how accurate would a test of drug response need to be to achieve cost-effective improvements in quality of life?

2. Journal Name: The European Journal of Health Economics

3. List of authors

Name	Preferred degree	Affiliation	Contact details
Huajie Jin<br>*(1st and Corresponding author)*	MSc	King's Health Economics (KHE), Institute of Psychiatry, Psychology & Neuroscience at King's College London, London, UK.	**Email**: huajie.jin@kcl.ac.uk<br>**Telephone:** +44 (0)20 7848 0878<br>**Address:**<br>King's Health Economics<br>Institute of Psychiatry, Psychology & Neuroscience at King's College London<br>Box 024, The David Goldberg Centre<br>SE5 8AF<br>London, UK
Paul McCrone	PhD	As above	**Address:**<br>King's Health Economics<br>Institute of Psychiatry, Psychology & Neuroscience at King's College London<br>Box 024, The David Goldberg Centre<br>SE5 8AF<br>London, UK
James H MacCabe<br>*(Last author)*	FRCPsych PhD	The Department of Psychosis Studies, Institute of Psychiatry, Psychology & Neuroscience at King's College London, London, UK.	**Address:**<br>The Department of Psychosis Studies, PO63<br>Institute of Psychiatry, Psychology & Neuroscience at King's College London<br>SE5 8AF<br>London, UK

Supplementary material

This supplementary material contains two appendices. Online Resource 1 reports the key input data used in the model, while Online Resource 2 reports the results of sensitivity analysis.

Online Resource 1. Description of input data

This section describes the key input data used in the model, including clinical data (section 1.1), cost and resource use data (section 1.2), and health-adjusted quality of life (HRQoL) data (section 1.3). A summary of all parameters used in the model, including their fixed values, ranges, distributions and sources, is reported in Table 1.

1.1 Clinical data

Diagnostic efficacy of stratified test. In the base-case analysis, both sensitivity and specificity of the stratified test were set at 60%. A range of 0-100% was tested using one-way sensitivity analyses.

Response to different antipsychotics in misclassified individuals. It is assumed that none of the false positive patients (i.e. clozapine responders or non-responders who are wrongly predicted to respond to a second-line conventional antipsychotic), will respond to any second-line antipsychotic. For those false negative patients (those would-be AP2 responders wrongly predicted not to respond), it was estimated that they would have a 71.16% probability of responding to clozapine and therefore remain on it [1]. A range of 0-1 was tested in two-way sensitivity analysis.

Probability of non-adherence. The probabilities of non-adherence to conventional antipsychotics were obtained from a network meta-analysis conducted for the NICE schizophrenia guideline 2014 [2]. The probability of all-cause discontinuation for clozapine-takers was obtained from Legge et al [3].

Probability of relapse for patients who didn't adhere to antipsychotics. The annual probability of relapse for patients who don't adhere to a conventional antipsychotic treatment was obtained from Mayoral-van et al 2016 [4]. The probability of relapse for patients who don't adhere to clozapine was obtained from Meltzer et al [5], as this paper reported separate relapse data for patients who were treatment responsive and resistant.

Baseline mortality rate. To calculate the mortality rate for people in this study, the age-specific standardised mortality ratio (SMR) observed in people with first episode psychosis [6] was multiplied by the age-and gender-specific mortality rates for the general population in England and Wales, as reported by the Office for National Statistics 2016 [7]. Mortality was calculated on the basis that the study population had a male to female ratio of 1.4 to 1 [8] and presented their first psychotic episode around 22 years of age (95% CI: 19-25 years) [9].

Impact of clozapine on mortality. Quite a few recent large-scale epidemiological studies have shown that use of clozapine is associated with reduced all-cause mortality and suicidality for schizophrenia patients [10-14]. A list of all epidemiological studies considered for this model is presented in Table 2. Although none of those studies have specified whether their patient sample is of treatment-resistant schizophrenia (TRS) or not except Wimberley et al [14], it was assumed that the vast majority of their patient sample would be TRS, as the use of clozapine is restricted to TRS in most countries. Of those epidemiological studies presented in Table 2, Wimberley's study [14] was considered most appropriate for use in our model because firstly, it was the latest study with the longest follow-up (up to 17 years); secondly, the results of Wimberley et al is comparable to other published studies. According to Wimberley et al [14], the adjusted hazard ratio for TRS who are currently on clozapine versus TRS who are not currently on clozapine is 0.53 (0.33-0.86). However, Wimberley's study [14] did not report separate mortality data for TRS patients who respond to clozapine, and TRS who do not respond to clozapine. For this model, it was assumed that clozapine is only effective in reducing all-cause mortality in TRS patients who respond to clozapine. Based on the assumption that 75% patients of TRS patients are clozapine responders and 25% are non-responders [15], it was calculated that the hazard ratio of all-cause mortality for clozapine responders on clozapine is 0.37 (95% CI: 0.11-0.81). Since there is a lack of evidence about the mortality rate of AP2 responders on clozapine, it is assumed that the use of clozapine does not have any impact on the all-cause mortality rate for AP2 responders.

Side effects of antipsychotics. The choice of side effects for consideration in the economic analysis was based on a number of criteria, including the number of people affected in the study population, the impact of side effects on health-related quality of life (HRQoL), the magnitude of costs incurred by their management and the availability of respective clinical data specific to the treatment options assessed. Based on the above criteria, four side effects of antipsychotics were modelled: weight gain, acute extrapyramidal symptoms (EPS), diabetes and neutropenia (for clozapine-users only). The probability data for weight gain, acute EPS and diabetes were

derived from the NICE guideline systematic review [2] and other published studies [16, 17]. The probability of developing neutropenia for patients on clozapine was obtained from Munro et al [18], which is a large-scale cohort study following 12,760 clozapine-users for up to 7.6 years.

1.2 Cost and resource use data

The model takes the perspective of the NHS and Personal Social Services (PSS), as recommended by NICE [19]. The financial year is 2016. The total costs for the treatment strategies were estimated by multiplying the unit costs with resource quantities. Unit costs were based on the NHS reference costs 2016-17 [20], prescription cost analysis (England) 2017 [21] or the Unit Costs of Health and Social Care 2017 [22]. The unit cost of the stratified test is assumed to be £500 per patient (a conservative estimate of the cost of a magnetic resonance spectroscopy scan) [23], with a range of £100-1000 tested in sensitivity analyses. Resources quantities were informed by the NICE schizophrenia guideline 2014 [2] and clinician's estimates where these were unavailable.

1.3 Health-related quality of life (HRQoL) data

HRQoL data was expressed as utilities from which quality adjusted life years (QALYs) were derived. Utility weights are usually elicited on a 0-1 ratio scale of 0 (death) to 1 (perfect health). The average utilities, for patients in different health states were taken from a UK study which reported separate utility data for stable or relapsed schizophrenia patients with or without adverse events associated with antipsychotic use [24].

Table 1. Summary of parameters used in model: base-line deterministic values, range used in one-way or two-way sensitivity analysis, distribution used in probabilistic sensitivity analysis, and references

Parameters	Base-line value	Range tested in one-way or two-way sensitivity analysis	Distribution	Source
**Diagnostic efficacy of predictive test**				
Sensitivity (proportion of second-line antipsychotic responders that are correctly identified as such)	0.60	0-1.00	Assumed fixed	Estimate of what may be achievable
Specificity (proportion of second-line antipsychotic non-responders that are correctly identified as such)	0.60	0-1.00	Assumed fixed	Estimate of what may be achievable
**Patient's demographic factors**				
Gender ratio for schizophrenia patients (male to female)	1.40	N/A	Assumed fixed	McGrath et al [8]
Age of presentation of first psychotic episode				
18-24 years	29.55%	N/A	Assumed fixed	Coid et al [25]
25-34 years	42.77%	N/A	Assumed fixed	As above
35-44 years	16.12%	N/A	Assumed fixed	As above
45-54 years	8.06%	N/A	Assumed fixed	As above
55-64 years	3.51%	N/A	Assumed fixed	As above
**Market share of different non-clozapine antipsychotics**				
Olanzapine	50.57%	N/A	Dirichlet distribution (n^1=44,786,709)	Calculated from Prescription cost analysis [21]
Amisulpride	7.30%	N/A	Dirichlet distribution (n^1=6,463,921)	As above
Aripiprazole	16.12%	N/A	Dirichlet distribution (n^1=14,280,763)	As above
Paliperidone	0.02%	N/A	Dirichlet distribution (n^1=17,712)	As above
Risperidone	18.84%	N/A	Dirichlet distribution (n^1=16,687,517)	As above
Haloperidol	4.27%	N/A	Dirichlet distribution (n^1=3,781,443)	As above
Flupenthixol decanoate	2.87%	N/A	Dirichlet distribution (n^1=2,541,626)	As above
**Distribution of patients who failed a first-line antipsychotic by subsequent response/non-response**				
Clozapine responder	62.50%	0%-79.15%	Dirichlet distribution (n=21.0)	Agid et al [15]
AP2 responder	16.67%	N/A	Dirichlet distribution (n=5.6)	As above
Clozapine non-responder	20.83%	N/A	Dirichlet distribution (n=7.0)	As above
**Response to different antipsychotics in misclassified individuals**				
AP2 responder's response to clozapine	71.16%	0-1	Beta distribution (SD assumed to be 50% of mean value)	Calculated from Agid et al[15] and McEvoy et al [1]
AP2 non-responder response to second-line antipsychotics	0%	N/A	Assumed fixed	Estimate
**Annual probability of discontinuing conventional antipsychotics because of non-adherence**				

Parameters	Base-line value	Range tested in one-way or two-way sensitivity analysis	Distribution	Source
Olanzapine	0.2730	0.1761-0.3848	Beta distribution (SD: 0.2140)	Calculated from NICE schizophrenia guideline [2]
Amisulpride	0.2435	0.1761-0.3848	Beta distribution (SD: 0.2088)	As above
Aripiprazole	0.3520	0.1761-0.3848	Beta distribution (SD: 0.2300)	As above
Paliperidone	0.3848	0.1761-0.3848	Beta distribution (SD: 0.2367)	As above
Risperidone	0.1761	0.1761-0.3848	Beta distribution (SD: 0.1799)	As above
Haloperidol	0.2516	0.1761-0.3848	Beta distribution (SD: 0.2093)	As above
Clozapine (year 1)	0.0570	0-0.4000	Beta distribution (SD assumed to be 50% of mean value)	Legge et al [3]
Clozapine (year 2 onwards)	0.0355	0-0.4000	As above	As above
**Probability of relapse for patients discontinue antipsychotic treatment due to non-adherence**				
Annual probability of relapse, first year following discontinuation of conventional antipsychotics	0.5650	0-0.8000	Beta distribution (SD assumed to be 50% of mean value)	Mayoral-van et al [4]
Annual probability of relapse, second year following discontinuation of conventional antipsychotics	0.2000	0-0.8000	As above	As above
Annual probability of relapse, year 3 onwards following discontinuation of conventional antipsychotics	0.0632	0-0.8000	As above	As above
Annual probability of relapse, following discontinuation of clozapine, for treatment response patients	0.7895	0-0.8000	Beta distribution (SD: 0.0935)	Meltzer et al [5]
Annual probability of relapse, following discontinuation of clozapine, for treatment resistant patients	0.3281	0-0.8000	Beta distribution (SD: 0.0587)	As above
**Annual probability of developing side effect - weight gain** *(assume that weight gain only happens on the first year of initiation of a particular antipsychotic)*				
Annual probability of weight gain for patients on haloperidol	0.2000	0.1716-0.4307	Beta distribution (α=31, β=124)	Calculated from NICE schizophrenia guideline [2]
Annual probability of weight gain for patients on flupentixol decanoate	0.2000	0.1716-0.4307	Beta distribution (α=31, β=124)	As above
OR of weight gain (Olanzapine v.s Haloperidol)	2.8631	1.7050-4.5090	Triangular distribution (min= 1.7050, max=4.5090)	As above
OR of weight gain (Amisulpride v.s Haloperidol)	1.8604	0.7345-4.0360	Triangular distribution (min= 0.7345, max=4.0360)	As above
OR of weight gain (Aripiprazole v.s Haloperidol)	0.7373	0.3498-1.3990	Triangular distribution (min= 0.3498, max=1.3990)	As above
OR of weight gain (Paliperidone v.s Haloperidol)	1.0779	0.4405-2.1640	Triangular distribution (min= 0.4405,	As above

Parameters	Base-line value	Range tested in one-way or two-way sensitivity analysis	Distribution	Source
			max=2.1640)	
OR of weight gain (Risperidone v.s Haloperidol)	1.0895	0.5214-2.0850	Triangular distribution (min= 0.5214, max=2.0850)	As above
RR of weight gain (Clozapine v.s Olanzapine)	1.5385	1.0000-2.0000	Triangular distribution (assumed min=1.0000, max=2.0000)	Calculated from McEvoy et al [1]
**Annual probability of developing side effects - acute EPS** *(first year of initiation of a particular antipsychotic)*				
Annual probability of EPS (Haloperidol)	0.5367	0.2366-0.5367	Beta distribution (α=928, β=801)	Calculated from NICE schizophrenia guideline [2]
Annual probability of EPS (Flupentixol decanoate)	0.4891	0.2366-0.5367	Beta distribution (α=45, β=47)	As above
OR of EPS (Olanzapine v.s Haloperidol)	0.2631	0.1832-0.3641	Triangular distribution (min=0.1832, max=0.3641)	As above
OR of EPS (Amisulpride v.s Haloperidol)	0.3993	0.2587-0.5836	Triangular distribution (min=0.2587, max=0.5836)	As above
OR of EPS (Aripiprazole v.s Haloperidol)	0.2517	0.1505-0.4002	Triangular distribution (min=0.1505, max=0.4002)	As above
OR of EPS (Paliperidone v.s Haloperidol)	0.2983	0.1179-0.6214	Triangular distribution (min=0.1179, max=0.6214)	As above
OR of EPS (Risperidone v.s Haloperidol)	0.4743	0.3680-0.5994	Triangular distribution (min=0.3680, max=0.5994)	As above
RR of EPS (Clozapine v.s Olanzapine)	0.3880	0.2000-0.6000	Triangular distribution (assumed min=0.2, max=0.6)	Calculated from Davies et al [17]
**Probability of developing side effect - acute EPS** *(following years)*				
Annual probability for all antipsychotics	Assumed 10% of first year estimate	N/A	N/A (no distribution assigned)	N/A
**Probability of developing side effect - diabetes** *(first year of initiation of a particular antipsychotic)*				
Haloperidol	0.0200	0.0156-0.0417	Beta distribution (α=2, β=98)	As above
Flupentixol decanoate	0.0200	0.0156-0.0417	Beta distribution (α=2, β=98)	As above
Olanzapine	0.0417	0.0156-0.0417	Beta distribution (SD assumed to be 50% of mean value)	As above
Amisulpride	0.0317	0.0156-0.0417	As above	As above
Aripiprazole	0.0156	0.0156-0.0417	As above	As above
Paliperidone	0.0212	0.0156-0.0417	As above	As above
Risperidone	0.0214	0.0156-0.0417	As above	As above

Parameters	Base-line value	Range tested in one-way or two-way sensitivity analysis	Distribution	Source
RR of diabetes (Clozapine vs Olanzapine)	1.2880	1.0000-2.0000	Triangular distribution (assumed min=0, max=0.6000)	Calculated from Davies et al [17]
**Annual probability of developing side effect - neutropenia**				
Clozapine	0.0125	0-0.0300	Triangular distribution (assumed min=0, max=0.0300)	Calculated from Munro et al [18]
**Annual mortality rates for male population (Death per 1,000 population)**				
15-19 years	0.30	N/A	Assumed fixed	Office for National Statistics [7]
20-24 years	0.50	N/A	Assumed fixed	As above
25-29 years	0.60	N/A	Assumed fixed	As above
30-34 years	0.80	N/A	Assumed fixed	As above
35-39 years	1.20	N/A	Assumed fixed	As above
40-44 years	1.70	N/A	Assumed fixed	As above
45-49 years	2.50	N/A	Assumed fixed	As above
50-54 years	3.70	N/A	Assumed fixed	As above
55-59 years	5.90	N/A	Assumed fixed	As above
60-64 years	9.60	N/A	Assumed fixed	As above
65-69 years	14.30	N/A	Assumed fixed	As above
70-74 years	24.50	N/A	Assumed fixed	As above
75-79 years	40.70	N/A	Assumed fixed	As above
80-84 years	73.20	N/A	Assumed fixed	As above
85 years+	162.40	N/A	Assumed fixed	As above
**Annual mortality rates for female population (Death per 1,000 population)**				
15-19 years	0.10	N/A	Assumed fixed	Office for National Statistics [7]
20-24 years	0.20	N/A	Assumed fixed	As above
25-29 years	0.30	N/A	Assumed fixed	As above
30-34 years	0.40	N/A	Assumed fixed	As above
35-39 years	0.70	N/A	Assumed fixed	As above
40-44 years	1.00	N/A	Assumed fixed	As above
45-49 years	1.60	N/A	Assumed fixed	As above
50-54 years	2.50	N/A	Assumed fixed	As above
55-59 years	4.00	N/A	Assumed fixed	As above
60-64 years	6.10	N/A	Assumed fixed	As above
65-69 years	9.40	N/A	Assumed fixed	As above
70-74 years	16.00	N/A	Assumed fixed	As above
75-79 years	28.10	N/A	Assumed fixed	As above
80-84 years	53.30	N/A	Assumed fixed	As above
85 years+	143.70	N/A	Assumed fixed	As above
**Standardized Mortality Ratios for all-cause of death (schizophrenia v.s general population)**				
16-29 years	7.40	N/A	Triangular distribution (min: 3.50, max: 15.50)	Reininghaus et al [6]
30-44 years	5.80	N/A	Triangular distribution (min: 3.70, max: 9.20)	As above

Parameters	Base-line value	Range tested in one-way or two-way sensitivity analysis	Distribution	Source
45-59 years	2.50	N/A	Triangular distribution (min: 1.20, max: 4.90)	As above
60-74 years	1.70	N/A	Triangular distribution (min: 0.80, max: 3.80)	As above
**Impact of clozapine on mortality (for clozapine-responders only)**				
Hazard ratio (HR) of all-cause mortality (clozapine v.s no clozapine)	0.37	0.11-0.81	Triangular distribution (min: 0.11, max: 0.81)	Calculated from Wimberley et al [14] based on the assumption that of all patients on clozapine, 75% of them are clozapine responders while 25% are non-responders
**Daily cost of antipsychotics**				
Olanzapine	£0.13	N/A	Assumed fixed	Calculated from Prescription cost analysis [21]
Amisulpride	£0.47	N/A	Assumed fixed	As above
Aripiprazole	£4.08	N/A	Assumed fixed	As above
Paliperidone	£6.58	N/A	Assumed fixed	As above
Risperidone	£0.36	N/A	Assumed fixed	As above
Haloperidol	£0.37	N/A	Assumed fixed	As above
Flupentixol decanoate	£0.24	N/A	Assumed fixed	As above
Clozapine	£1.56	N/A	Assumed fixed	As above
**Other cost data**				
Cost of predictive test	£500.00	£100.00-1,000.00	Gamma distribution (SD assumed to be 50% of mean value)	Estimate
Cost of blood test for clozapine	£2.65	N/A	Assumed fixed	Akhtar et al [26]
Cost of one attendance at clozapine clinic	£34.82	N/A	Assumed fixed	Resource use were obtained from the finance department of a clozapine clinic based at the South London and Maudsley NHS Foundation Trust
Cost of switching between antipsychotics	£426.00	N/A	Gamma distribution (SD assumed to be 50% of mean value)	Resource use was informed by the NICE schizophrenia guideline [2]. Unit cost was obtained from PSSRU[22]
Annual cost of treating weight gain for patients adhere to antipsychotics (Year 1)	£97.20	£0-1,000.00	As above	As above
Annual cost of treating weight gain for patients adhere to antipsychotics (Year 2 onwards)	£309.68	£0-1,000.00	As above	Calculated and uplifted from

Parameters	Base-line value	Range tested in one-way or two-way sensitivity analysis	Distribution	Source
				Scarborough et al [27]
Annual cost of treating weight gain for patients who discontinue antipsychotics due to non-adherence	50% of original cost	0-100% of original cost	As above	Estimate
Annual cost of treating acute EPS for patients adhere to antipsychotics	£51.95	£0-500.00	As above	Resource use was informed by the NICE schizophrenia guideline [2]. Unit cost was obtained from PSSRU [22]
Annual cost of treating acute EPS for patients discontinue antipsychotics due to non-adherence	£0	0-100% of original cost	As above	Estimate
Annual cost of treating diabetes with/out complications for patients adhere to antipsychotics	£1,336.31	£0-2,000.00	As above	Probability of developing complications of diabetes was informed by the NICE schizophrenia guideline [2]. Cost of treating each complication was uplifted from UKPDS [28]
Annual cost of treating diabetes with/out complications for patients who discontinue antipsychotics due to non-adherence	50% of original cost	0-100% of original cost	As above	Estimate
Cost of treating neutropenia	£469.48	£0-1,000.00	Gamma distribution (SD assumed to be 50% of mean value)	NHS reference cost 2016-17 [20]
Annual cost of treating patients with active psychosis	£39,141	N/A	As above	Uplifted from the NICE schizophrenia guideline [2]
Annual cost of treating remitted patients	£15,086	£10,000-£39,141	As above	As above
**Health-related quality of life data**				
Relapse	0.4790	0.1900-0.6040	Beta distribution (SD: 0.0330)	Briggs et al [24]
Stable schizophrenia without adverse events	0.8650	0.8650-0.9190	Beta distribution (SD: 0.0210)	As above
Stable schizophrenia with weight gain	0.7790	0.7790-0.8650	Beta distribution (SD: 0.0240)	As above
Stable schizophrenia with diabetes	0.7120	0.7120-0.8650	Beta distribution (SD: 0.0280)	As above
Stable schizophrenia with acute EPS	0.5740	0.5740-0.8650	Beta distribution (SD: 0.0320)	As above
**Other input data**				

Parameters	Base-line value	Range tested in one-way or two-way sensitivity analysis	Distribution	Source
Annual discount rate for both costs and outcomes	0.0350	0-0.050	Assumed fixed	NICE guideline manual [19]
Cycle length	1 year	N/A	Assumed fixed	Estimate
Number of cycles	80	10-100	Assumed fixed	Estimate

Notes:

1. N refers to the number of daily doses prescribed, calculated by dividing the total amount of net ingredients prescribed by the recommended daily dose.

Table 2. The effect of clozapine on all-cause mortality and suicidality reported by recent large-scale studies

Study	Publication year	Country	Basis of analysis	Population	Follow-up period	Effect of clozapine on mortality
**Tiihonen et al [10]**	2009	Finland	Nationwide registers in Finland	All patients in Finland (n=66,881) who were admitted with a diagnosis of schizophrenia	11-year	• All-cause mortality<br>Clozapine v.s Perphenazine: adjusted HR for clozapine (0.74, (95% CI: 0.60–0.91)). Clozapine v.s all other antipsychotics: P<0.0001
**Kiviniemi et al [12]**	2013	Finland	Four Finnish national registers.	All patients presenting with first onset of schizophrenia (n= 6,987)	5-year	• All-cause mortality<br>Clozapine v.s no antipsychotic: adjusted odds ratio: 0.35 (95% CI: 0.21-0.58, p<0.001)
**Hayes et al [11]**	2014	UK	A large, anonymized, electronic mental health Records database which cover 200,000 patients.	14,754 individuals with serious mental illness including schizophrenia (n= 9437), schizoaffective (n=805) and bipolar disorders (n=4,512) aged ≥ 15 years.	5-year	• All-cause mortality<br>Prescribed clozapine v.s Not prescribed clozapine: adjusted HR for clozapine (0.4; 95% CI 0.2–0.7; p = 0.001)<br>• Likelihood for suicide<br>Prescribed clozapine v.s Not prescribed clozapine: adjusted HR for clozapine (0.29; 95% CI: 0.14–0.63; p = 0.002)
**Weitoft et al [13]**	2014	Sweden	National Patient Register, the Swedish Prescribed Drug Register and the National Cause of Death Register.	all patients (n=26,046) in Sweden who had been treated for schizophrenia from 2006 to 2009.	3-month	• All-cause mortality<br>Prescribed clozapine v.s haloperidol, adjusted odds ratio: 0.92 (95% CI: 0.70–1.22)<br>• Death by suicide<br>Prescribed clozapine v.s haloperidol, adjusted odds ratio: 0.45 (95% CI 0.20–0.98)<br>• Attempted suicide<br>Prescribed clozapine v.s haloperidol, adjusted odds ratio: 0.44 (0.28–0.70)
**Wimberley et al [14]**	2017	EU	The Danish Psychiatric Central Register, National Patient Registry, Civil Registration System and the National Prescription Registry.	2,370 individuals meeting criteria for treatment-resistant schizophrenia after 1996 and followed until death and first episode of suicidal behaviour, emigration, or June 1, 2013.	17-year	• All-cause mortality<br>Current clozapine v.s No current clozapine, adjusted hazard ratio: 0.53 (0.33-0.86)

Online Resource 2. Results of sensitivity analysis

This appendix reports results of one-way and two-way sensitivity analysis in Section 2.1 and 2.2, respectively.

2.1 One-way sensitivity analysis results

The conclusion of the base case analysis (SMA being the most cost-effective strategy) was robust to all scenarios tested. The detailed results of one-way sensitivity analysis are reported in Table 3.

Table 3. One-way sensitivity analyses results

Analysis	Value tested	Cost savings of SMA compared to TAU (£)	QALY gains of SAM compared to TAU	Conclusion[1]
**Clinical parameters**				
Annual probability of discontinuing antipsychotics because of non-adherence, for patients on conventional antipsychotics	0.1761	7,037	0.1008	SMA dominant
	0.3848	7,659	0.1064	SMA dominant
Annual probability of discontinuing antipsychotics because of non-adherence, for patients on clozapine (year 1)	0	7,480	0.1053	SMA dominant
	0.40	6,308	0.0891	SMA dominant
Annual probability of discontinuing antipsychotics because of non-adherence, for patients on clozapine (year 2 onwards)	0	8,289	0.1181	SMA dominant
	0.40	2,395	0.0290	SMA dominant
Probability of relapse following discontinuation of conventional antipsychotics (year 1)	0	6,309	0.0792	SMA dominant
	0.80	8,146	0.1220	SMA dominant
Probability of relapse following discontinuation of conventional antipsychotics (year 2)	0	7,122	0.0982	SMA dominant
	0.80	8,362	0.1270	SMA dominant
Annual probability of relapse following discontinuation of conventional antipsychotics (year 3 onwards)	0	6.490	0.0836	SMA dominant
	0.80	8,564	0.1317	SMA dominant
Annual probability of relapse, following discontinuation of clozapine, for treatment response patients	0	8,645	0.1404	SMA dominant
	0.80	7,360	0.1037	SMA dominant
Annual probability of relapse, following discontinuation of clozapine, for treatment resistant patients	0	7,587	0.1038	SMA dominant
	0.80	7,345	0.1036	SMA dominant
Probability of developing weight gain for patients on haloperidol or flupentixol decanoate	0.1716	7,383	0.1100	SMA dominant
	0.4307	7,249	0.0675	SMA dominant
OR of weight gain (Olanzapine v.s Haloperidol)	1.7050	7,421	0.1216	SMA dominant
	4.5090	7,307	0.0867	SMA dominant
OR of weight gain (Amisulpride v.s Haloperidol)	0.7345	7,361	0.1033	SMA dominant
	4.0360	7,365	0.1043	SMA dominant
OR of weight gain (Aripiprazole v.s Haloperidol)	0.3498	7,361	0.1033	SMA dominant
	1.3990	7,365	0.1043	SMA dominant
OR of weight gain (Paliperidone v.s Haloperidol)	0.4405	7,363	0.1038	SMA dominant
	2.1640	7,363	0.1038	SMA dominant
OR of weight gain (Risperidone v.s Haloperidol)	0.5214	7,360	0.1030	SMA dominant
	2.0850	7,367	0.1047	SMA dominant
RR of weight gain (Clozapine v.s Olanzapine)	1.0000	7,449	0.1290	SMA dominant
	2.0000	7,293	0.0832	SMA dominant
Annual probability of developing acute EPS for patients on haloperidol or flupentixol decanoate, first year of initiation of a particular antipsychotic	0.2366	7,362	0.0994	SMA dominant
	0.5367	7,363	0.1038	SMA dominant
OR of EPS (Olanzapine v.s Haloperidol)	0.1832	7,363	0.1038	SMA dominant

	0.3641	7,363	0.1037	SMA dominant
OR of EPS (Amisulpride v.s Haloperidol)	0.2587	7,363	0.1035	SMA dominant
	0.5836	7,363	0.1041	SMA dominant
OR of EPS (Aripiprazole v.s Haloperidol)	0.1505	7,363	0.1032	SMA dominant
	0.4002	7,363	0.1044	SMA dominant
OR of EPS (Paliperidone v.s Haloperidol)	0.1179	7,363	0.1038	SMA dominant
	0.6214	7,363	0.1038	SMA dominant
OR of EPS (Risperidone v.s Haloperidol)	0.3680	7,363	0.1033	SMA dominant
	0.5994	7,363	0.1043	SMA dominant
RR of EPS (Clozapine v.s Olanzapine)	0.2000	7,363	0.1066	SMA dominant
	0.6000	7,362	0.1006	SMA dominant
Annual probability of developing diabetes for patients on conventional antipsychotics	0.0156	7,348	0.1027	SMA dominant
	0.0417	7,372	0.1044	SMA dominant
RR of diabetes (Clozapine v.s Olanzapine)	1.0000	7,383	0.1061	SMA dominant
	2.0000	7,312	0.0980	SMA dominant
Annual probability of developing neutropenia for patients on clozapine	0	7,377	0.1038	SMA dominant
	0.0300	7,359	0.1038	SMA dominant
Hazard ratio of all-cause mortality (clozapine v.s no clozapine)	0.1100	7,204	0.1111	SMA dominant
	0.8100	7,607	0.0925	SMA dominant
**Cost parameters**				
Cost of the stratified test	£100	7,763	0.1038	SMA dominant
	£1,000	6,863	0.1038	SMA dominant
Annual cost of treating weight gain for patients adhere to antipsychotics	£0	7,553	0.1038	SMA dominant
	£1,000	6,932	0.1038	SMA dominant
Annual cost of treating weight gain for patients who discontinue antipsychotics due to non-adherence	0	7,345	0.1038	SMA dominant
	Same as patients who didn't discontinue antipsychotics	7,400	0.1038	SMA dominant
Annual cost of treating acute EPS for patients adhere to antipsychotics	£0	7,362	0.1038	SMA dominant
	£500.00	7,373	0.1038	SMA dominant
Annual cost of treating acute EPS for patients discontinue antipsychotics due to non-adherence	0	7,363	0.1038	SMA dominant
	Same as patients who didn't discontinue antipsychotics	7,371	0.1038	SMA dominant
Annual cost of treating diabetes with/out complications for patients adhere to antipsychotics	£0	7,427	0.1038	SMA dominant
	£2,000.00	7,331	0.1038	SMA dominant
Annual cost of treating diabetes with/out complications for patients who discontinue antipsychotics due to non-adherence	0	7,354	0.1038	SMA dominant
	Same as patients who didn't discontinue antipsychotics	7,372	0.1038	SMA dominant
Cost of treating neutropenia per episode	£0	7,377	0.1038	SMA dominant
	£1,000.00	7,347	0.1038	SMA dominant

Annual cost of treating remitted patients	£15,656 (40% of treatment cost for relapsed patients)	7,134	0.1038	SMA dominant
	£23,484 (60% of treatment cost for relapsed patients)	3,986	0.1038	SMA dominant
	£31,313 (80% of treatment cost for relapsed patients)	837	0.1038	SMA dominant
	£37,184 (100% of treatment cost for relapsed patients)	-1,524	0.1038	SMA is more cost-effective (ICER for SMA=14,683 per QALY, which is less than NICE's threshold of £20,000 per QALY)
**Utility parameters**				
Utility for schizophrenia patients in relapse	0.1900	7,363	0.2143	SMA dominant
	0.6040	7,363	0.0559	SMA dominant
Utility for schizophrenia patients in remission without side effects	0.8650	7,363	0.1038	SMA dominant
	0.9190	7,363	0.0866	SMA dominant
Utility for schizophrenia patients in remission with weight gain	0.7790	7,363	0.1038	SMA dominant
	0.8650	7,363	0.1630	SMA dominant
Utility for schizophrenia patients in remission with diabetes	0.7120	7,363	0.1038	SMA dominant
	0.8650	7,363	0.1122	SMA dominant
Utility for schizophrenia patients in remission with EPS	0.5740	7,363	0.1038	SMA dominant
	0.8650	7,363	0.0969	SMA dominant
**Other parameters**				
No. of cycles	10	7,463	0.1076	SMA dominant
	100	7,363	0.1038	SMA dominant
Discount rate for both cost and QALYs	0	7,391	0.1040	SMA dominant
	0.05	7,287	0.1031	SMA dominant

Notes:

1. There are two scenarios under which SMA could be considered to be cost-effective:

- Scenario 1. Compared with TAU, SMA is less costly and more clinically effective. In this case, SMA 'dominates' TAU and no further justification is necessary.
- Scenario 2. Compared with TAU, SMA is more effective, but also more expensive. In this case, the decision whether to implement the SMA would depend on how much the payer of healthcare (the NHS in the UK) is prepared to pay per additional unit of QALY. The incremental cost-effectiveness ratio (ICER) is the ratio of the difference in cost divided by the difference in QALYS and is expressed in UK pounds per additional QALY. In line with the NICE reference case, a willingness-to-pay (WTP) threshold of £20,000 per additional QALY was used. Thus, if the ICER of SMA versus TAU is less than £20,000 per QALY, then SMA is more cost-effective than TAU.

2.2 Two-way sensitivity analysis results

The results of two-way sensitivity analysis are presented in Figure 1 below. Figure 1A reports the combined effects of sensitivity and specificity of the stratifier on the results. It shows that:

- If the sensitivity of the stratifier is 0%, as long as the specificity of the test is no less than 11.50%, SMA is more cost-effective than TAU
- If the sensitivity of the stratifier is 50.00%, as long as the specificity of the test is no less than 6.00%, SMA is more cost-effective than TAU

Figure 1B reports the combined effects of: proportion of clozapine responder in patients who failed a first-line antipsychotic, and proportion of AP2 responders who respond to clozapine. The results show that:

- If 50% of the AP2 responders would respond to clozapine, as long as the proportion of clozapine responder is no less than 23.75%, SMA is more cost-effective than TAU
- If none of the AP2 responders would respond to clozapine, as long as the proportion of clozapine responder is no less than 35.62%, SMA is more cost-effective than TAU

Figure 1. Two-way sensitivity analysis result showing the most cost-effective strategy [a]

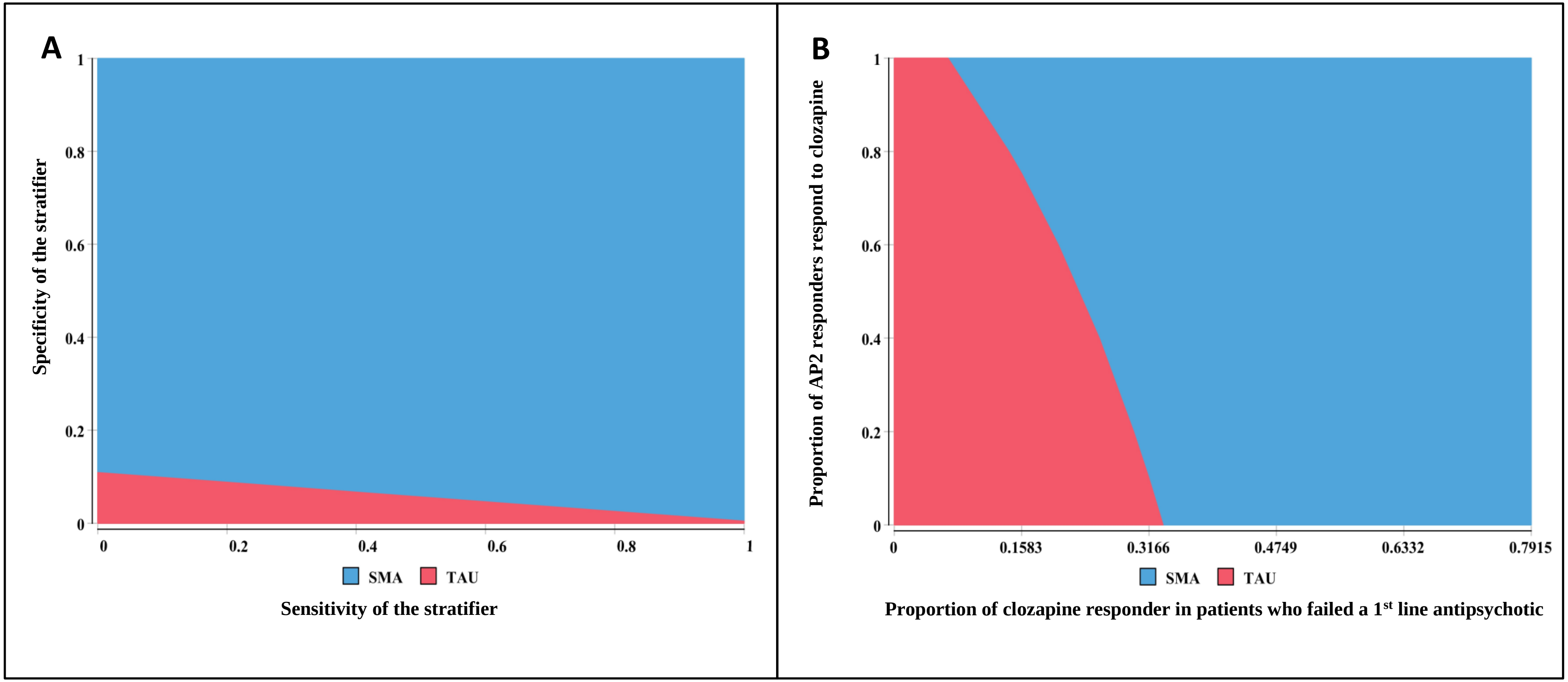

Footnote:
a: Based on a willingness-to-pay threshold of £20,000 per additional unit of QALY.
Abbreviation:

- SMA: Stratified medicine algorithm
- TAU: Treatment as usual

Reference

1. McEvoy JP, Lieberman JA, Stroup TS, Davis SM, Meltzer HY, Rosenheck RA et al. Effectiveness of clozapine versus olanzapine, quetiapine, and risperidone in patients with chronic schizophrenia who did not respond to prior atypical antipsychotic treatment. The American journal of psychiatry. 2006;163(4):600-10. doi:10.1176/appi.ajp.163.4.600.
2. National Collaborating Centre for Mental Health. Psychosis and schizophrenia in adults. NICE guideline (CG178). 2014.
3. Legge SE, Hamshere M, Hayes RD, Downs J, O'Donovan MC, Owen MJ et al. Reasons for discontinuing clozapine: A cohort study of patients commencing treatment. Schizophr Res. 2016. doi:10.1016/j.schres.2016.05.002.
4. Mayoral-van Son J, de la Foz VO, Martinez-Garcia O, Moreno T, Parrilla-Escobar M, Valdizan EM et al. Clinical outcome after antipsychotic treatment discontinuation in functionally recovered first-episode nonaffective psychosis individuals: a 3-year naturalistic follow-up study. J Clin Psychiatry. 2016;77(4):492-500.
5. Meltzer HY, Lee MA, Ranjan R, Mason EA, Cola PA. Relapse following clozapine withdrawal: effect of neuroleptic drugs and cyproheptadine. Psychopharmacology. 1996;124(1-2):176-87.
6. Reininghaus U, Dutta R, Dazzan P, Doody GA, Fearon P, Lappin J et al. Mortality in schizophrenia and other psychoses: A 10-year follow-up of the AEsOP first-episode cohort. Schizophrenia Bulletin. 41 (3) (pp 664-673), 2015. Date of Publication: 2015.; 2015.
7. Office for National Statistics. Comparison of mortality rates by age and sex in 1963 and 2013. London: Office for National Statistics; 2016.
8. McGrath JJ. Variations in the incidence of schizophrenia: Data versus dogma. Schizophrenia Bulletin. 32 (1) (pp 195-197), 2006. Date of Publication: January 2006.; 2006.
9. Kessler RC, Amminger GP, Aguilar-Gaxiola S, Alonso J, Lee S, Ustun TB. Age of onset of mental disorders: A review of recent literature. Current Opinion in Psychiatry. 20 (4) (pp 359-364), 2007. Date of Publication: July 2007.; 2007.
10. Tiihonen J, Lonnqvist J, Wahlbeck K, Klaukka T, Niskanen L, Tanskanen A et al. 11-year follow-up of mortality in patients with schizophrenia: a population-based cohort study (FIN11 study). The Lancet. 374 (9690) (pp 620-627), 2009. Date of Publication: 28 Aug 2009.; 2009.
11. Hayes RD, Downs J, Chang CK, Jackson RG, Shetty H, Broadbent M et al. The effect of clozapine on premature mortality: An assessment of clinical monitoring and other potential confounders. Schizophrenia Bulletin. 2014;41(3):644-55.
12. Kiviniemi M, Suvisaari J, Koivumaa-Honkanen H, Hakkinen U, Isohanni M, Hakko H. Antipsychotics and mortality in first-onset schizophrenia: prospective Finnish register study with 5-year follow-up. Schizophrenia Research. 2013;150(1):274-80.
13. Ringback Weitoft G, Berglund M, Lindstrom EA, Nilsson M, Salmi P, Rosen M. Mortality, attempted suicide, re-hospitalisation and prescription refill for clozapine and other antipsychotics in Sweden-a register-based study. Pharmacoepidemiology & Drug Safety. 2014;23(3):290-8.
14. Wimberley T, MacCabe JH, Laursen TM, Sorensen HJ, Astrup A, Horsdal HT et al. Mortality and Self-Harm in Association With Clozapine in Treatment-Resistant Schizophrenia. The American journal of psychiatry. 2017;174(10):990-8. doi:10.1176/appi.ajp.2017.16091097.
15. Agid O, Arenovich T, Sajeev G, Zipursky RB, Kapur S, Foussias G et al. An algorithm-based approach to first-episode schizophrenia: Response rates over 3 prospective antipsychotic trials with a retrospective data analysis. Journal of Clinical Psychiatry. 72 (11) (pp 1439-1444), 2011. Date of Publication: November 2011.; 2011.
16. Hagg S, Joelsson L, Mjorndal T, Spigset O, Oja G, Dahlqvist R. Prevalence of diabetes and impaired glucose tolerance in patients treated with clozapine compared with patients treated with conventional depot neuroleptic medications. Journal of Clinical Psychiatry. 59 (6) (pp 294-299), 1998. Date of Publication: June 1998.; 1998.

17. Davies A, Vardeva K, Loze JY, L'Italien GJ, Sennfalt K, Van Baardewijk M. Cost-effectiveness of atypical antipsychotics for the management of schizophrenia in the UK. Current Medical Research and Opinion. 24 (11) (pp 3275-3285), 2008. Date of Publication: 2008.; 2008.
18. Munro J, O'Sullivan D, Andrews C, Arana A, Mortimer A, Kerwin R. Active monitoring of 12760 clozapine recipients in the UK and Ireland: Beyond pharmacovigilance. British Journal of Psychiatry. 175 (DEC.) (pp 576-580), 1999. Date of Publication: 1999.; 1999.
19. National Institute for Health and Care Excellence. Developing NICE guidelines: the manual. 2014.
20. Department of Health. NHS reference costs 2016 to 2017. 2017.
21. Prescription Cost Analysis. Prescribing & Medicines Team Health and Social Care Information Centre. Prescription Cost Analysis. 2017.
22. Curtis L. Unit Costs of Health & Social Care 2017. Canterbury: Personal social services research unit, University of Kent; 2017.
23. Mouchlianitis E, Bloomfield MAP, Law V, Beck K, Selvaraj S, Rasquinha N et al. Treatment-Resistant Schizophrenia Patients Show Elevated Anterior Cingulate Cortex Glutamate Compared to Treatment-Responsive. Schizophrenia Bulletin. 2016;42(3):744-52. doi:10.1093/schbul/sbv151.
24. Briggs A, Wild D, Lees M, Reaney M, Dursun S, Parry D et al. Impact of schizophrenia and schizophrenia treatment-related adverse events on quality of life: Direct utility elicitation. Health and Quality of Life Outcomes. 6 (no pagination), 2008. Article Number: 105. Date of Publication: 2008.; 2008.
25. Coid JW, Kirkbride JB, Barker D, Cowden F, Stamps R, Yang M et al. Raised incidence rates of all psychoses among migrant groups: findings from the East London first episode psychosis study. Arch Gen Psychiatry. 2008;65(11):1250-8.
26. Akhtar W, Chung Y. Saving the NHS one blood test at a time. BMJ Qual Improv Report 2014(2).
27. Scarborough P, Bhatnagar P, Wickramasinghe KK, Allender S, Foster C, Rayner M. The economic burden of ill health due to diet, physical inactivity, smoking, alcohol and obesity in the UK: an update to 2006-07 NHS costs. Journal of public health (Oxford, England). 2011;33(4):527-35. doi:10.1093/pubmed/fdr033.
28. Alva ML, Gray A, Mihaylova B, Leal J, Holman RR. The impact of diabetes-related complications on healthcare costs: new results from the UKPDS (UKPDS 84). Diabetic medicine : a journal of the British Diabetic Association. 2015;32(4):459-66. doi:10.1111/dme.12647.
